# The analgesic effects of intraoperative total intravenous anesthesia (TIVA) with propofol versus sevoflurane after colorectal surgery

**DOI:** 10.1097/MD.0000000000011615

**Published:** 2018-08-03

**Authors:** Stanley Sau Ching Wong, Siu Wai Choi, Yvonne Lee, Michael G. Irwin, Chi Wai Cheung

**Affiliations:** Department of Anaesthesiology, Queen Mary Hospital, Hong Kong, Hong Kong.

**Keywords:** acute, colorectal surgery, pain, postoperative analgesia, postoperative opioid consumption, propofol, sevoflurane, total intravenous anesthesia

## Abstract

Clinical studies have shown that total intravenous anesthesia (TIVA) with propofol is associated with better postoperative pain control compared with inhalational anesthesia, while other studies have not shown any benefit. The analgesic effect of TIVA with propofol in colorectal surgery has not been studied. The aim of this study is to evaluate the postoperative analgesic effects of TIVA with propofol versus inhalational sevoflurane in colorectal surgery.

This is a retrospective case–control study. Records of patients undergoing colorectal surgery from 2014 to 2016 (36 months) were retrieved. Ninety-five patients who received TIVA with propofol were matched against 95 patients who received inhalational sevoflurane. Acute postoperative numerical rating scale (NRS) pain scores, postoperative morphine consumption, patient satisfaction, and side effects were compared and analyzed for differences between TIVA with propofol and sevoflurane.

There were no significant differences in NRS pain scores, incidence of side effects, and patient satisfaction between the 2 groups. Patients receiving TIVA with propofol had significantly reduced total morphine consumption (*P* < .001), and daily morphine consumption on postoperative days 1 (*P* = .031), 2 (*P* = .002), and 3 (*P* = .031) compared with those receiving sevoflurane.

TIVA with propofol was not associated with improved postoperative analgesia, better patient satisfaction, or reduced side effects. It may reduce postoperative opioid consumption after colorectal surgery.

## 1 Introduction

Postoperative pain after surgery is still a significant problem, and poor control of postoperative pain can result in increased morbidity, prolonged recovery, reduced patient satisfaction, and increased incidence of chronic postsurgical pain.^[1,2]^ Analgesics that improve analgesia, reduce opioid use, and reduce opioid-related side effects can improve outcomes and enhance recovery.^[3]^

Propofol is one of the most commonly used intravenous anesthetic drugs both for induction and maintenance of general anesthesia. Advantages of total intravenous anesthesia (TIVA) with propofol also include reduced nausea and vomiting, reduced atmospheric pollution, and a better wake up profile.^[4]^ Propofol also has analgesic properties.^[5]^ In animal studies, propofol has been shown to reduce pro-inflammatory cytokine levels and inhibit activation of N-methyl-D-aspartate (NMDA) receptors.^[6,7]^ Clinical studies have shown that propofol provided improved acute postoperative analgesia compared with inhalational anesthesia.^[8–11]^ A meta-analysis of 14 clinical trials also found that propofol was associated with lower pain scores 24 hours after surgery.^[12]^ Furthermore, TIVA with propofol may reduce incidence of chronic postsurgical pain.^[10,13]^ However, other clinical studies have found no beneficial analgesic effect after surgery with propofol.^[14,15]^ As pain intensity and mechanism of pain is probably different with different types of surgery, the usefulness of propofol as an analgesic may vary in the context of different surgical procedures. This is in agreement with the concept of procedure-specific analgesia.^[16]^

Multimodal opioid-sparing analgesia is recommended for colorectal surgery and is a vital component of enhanced recovery after surgery (ERAS) programs.^[17]^ Reduced opioid use is associated with earlier return of bowel function and shorter length of hospital stay.^[18,19]^ Opioid-sparing analgesics that may be useful for colorectal surgery include paracetamol, selective and nonselective nonsteroidal anti-inflammatory analgesic drugs (NSAIDs and COX-2 inhibitors), alpha-2-delta ligands, intravenous lignocaine, and alpha 2-agonists.^[20–25]^ The analgesic properties of propofol may make it a useful opioid-sparing analgesic adjunct for colorectal surgery in the context of a multimodal analgesic regime. The analgesic effect of intraoperative maintenance with propofol versus inhalational agents specifically for colorectal surgery has not been evaluated before. The main aims of this study were to determine whether TIVA with propofol would improve postoperative analgesia and reduce opioid consumption after colorectal surgery. A retrospective case-controlled study was performed to compare the analgesic effects of TIVA with propofol versus inhalational sevoflurane. We hypothesized that TIVA with propofol would be associated with less postoperative pain and reduced postoperative opioid consumption.

## 2 Methods

The study was approved by the Institutional Review Board of Queen Mary Hospital and the University of Hong Kong. It was registered at ClinicalTrials.gov (NCT03058354). There was no requirement to obtain written informed consent from patients, as this study was retrospective in nature. The data were delinked from patient identifiers and anonymized before analysis so that none of the researchers were aware of patient identification. Records of patients after colorectal surgery and under the care of the acute pain service (APS) between January 1, 2014, and December 29, 2016, in Queen Mary hospital were reviewed and analyzed. Data collected included demographic data [age, body weight, gender, and American Society of Anesthesiologists (ASA) physical status]; types of colorectal surgery performed (open surgery or minimally invasive surgery, where laparoscopic or robotic surgery was considered as minimally invasive surgery); types of general anesthesia techniques (TIVA with propofol, inhalational anesthesia with sevoflurane); with or without intraoperative use of ketamine; pain intensity as verbal numerical rating scale (NRS, 0 = no pain, 10 = the worst imaginable pain) at rest and during coughing from postoperative days 1 to 3; daily, accumulative and total postoperative morphine consumption and duration of morphine patient-controlled analgesia (PCA) use; incidence of adverse events (nausea, vomiting, dizziness, pruritus, confusion), and patient satisfaction with pain relief. Exclusion criteria included the following: missing essential data, difficulty in assessment of postoperative pain (e.g., postoperative mechanical ventilation, language barriers), early termination of PCA morphine due to deterioration of patients’ condition, patients requiring a second operation, and patients participating in other research projects.

Patients did not receive premedication. Patients receiving inhalational sevoflurane anesthesia (SEVO group) were induced with 1 to 2 mg/kg of intravenous propofol, and then given a bolus of 1 μg/kg of remifentanil and 0.5 mg/kg of atracurium or 0.6 mg/kg of rocuronium before endotracheal intubation. General anesthesia was then maintained with sevoflurane, air, and oxygen, with sevoflurane level titrated to between 0.7 and 1 MAC. Patients receiving intravenous maintenance with propofol (TIVA group) were induced and maintained with a target-controlled infusion of propofol using the Marsh effect site model. Patients in the TIVA group also received 1 μg/kg bolus of remifentanil and 0.5 mg/kg of atracurium or 0.6 mg/kg of rocuronium before intubation. Patients from both groups received intraoperative remifentanil infusion titrated according to hemodynamic response up to a maximum of 0.2 μg/kg/min. Zero-point 1 mg/kg of intravenous morphine was given before surgical incision. An additional 0.1 mg/kg of morphine was given in divided doses at the discretion of the anesthetist for surgical procedures lasting more than 2 hours. Intravenous ketamine at a dose of 0.5 to 1 mg/kg was given at the discretion of the anesthetist. Up to 2 mL/kg of 0.5% levobupivicaine was infiltrated into the surgical wound by the surgeon during wound closure. Reversal of neuromuscular blockade was achieved with 0.02 mg/kg of atropine and 0.05 mg/kg of neostigmine.

Vital signs, including blood pressure, oxygen saturation (SpO2), heart rate, and body temperature, were monitored in the postanesthesia care unit (PACU). Two milligrams of intravenous morphine were given to patients if their verbal NRS was 4 or above. This was repeated every 5 minutes until their NRS was 3 or below. All patients were given intravenous PCA with morphine. The PCA morphine setting was 1 mg bolus, 5-minute lockout interval, maximum hourly dose limit of 0.1 to 0.15 mg/kg/hour, and without background morphine infusion. Rescue pain medication was prescribed in the form of subcutaneous or intramuscular morphine injection at a dose of 0.05 mg/kg every 4 hours as needed. Intravenous ondansetron 4 mg every 4 hours was prescribed for nausea and vomiting on an as needed basis.

Fluid diet was started on postoperative day 0 in the absence of obvious surgical complications. Regular oral analgesics consisting of tramadol 50 mg 3 times a day, paracetamol 1 g 3 times daily, and celecoxib 200 mg twice daily were started. The patients were assessed by an anesthetist from the APS team at least once a day. In addition, the APS team was informed if pain control was poor. PCA morphine settings could then be adjusted after assessment to give a larger bolus dose and higher hourly limit. NRS pain scores at rest and with coughing, daily PCA morphine consumption, and side effects were recorded every day until APS discharge. PCA morphine was stopped when NRS score was less than 3 during coughing; daily PCA morphine use was less than 0.1 mg/kg, or upon patient request. Oral analgesics and rescue analgesics were continued after cessation of PCA morphine. At the time of PCA morphine cessation, patient satisfaction regarding pain service was evaluated. Patients were asked to rate their satisfaction as “good,” “fair,” or “unsatisfactory.” Reasons for “unsatisfactory” grading were sought.

Calculation for the sample size was based on the effect from a previous study on analgesic efficacy for colorectal surgery using the primary outcome of pain at rest at 24 hours.^[26]^ The mean visual analogue scale (VAS) pain score of the treatment group was 1.8, while the mean VAS pain score of the control group was 2.7 with *P* = .002 and an estimated pooled standard deviation of 2.2. At a power of 0.80 and an alpha value of 0.05, 94 patients would be required in each group. As this study was retrospective in nature, all patients who underwent colorectal surgery between January 2014 and December 2016 were screened for eligibility. A total of 95 patients in each group who satisfied our matching criteria were included in this study from our database.

Each patient who received TIVA was matched 1:1 to patients who had received sevoflurane according to age, gender, ASA physical status, types of surgery performed (open or minimally invasive surgery), and with or without intraoperative use of ketamine.^[27]^ This 1:1 patient matching is a more comprehensive method than using the propensity score.^[28,29]^ Patient demographics are presented as means (SD) for parametric data, and percentage where appropriate. Differences in pain scores were tested for using unpaired *t* test with Welch correction, while Mann–Whitney test was used to look for differences in PCA morphine consumption after it was determined that the data did not follow a normal distribution. Differences in postoperative side effects and overall satisfaction were tested for using Fisher exact test. Statistical Package for the Social Sciences (SPSS Statistics version 20; IBM Corp., Armonk, NY) statistical software and GraphPad Prism version 7.00 for Windows, GraphPad Software, La Jolla California USA were used for data analysis.

## 3 Results

Seven hundred and twenty-one records of patients who underwent colorectal surgery under the management of the APS team were screened. It was found that 110 patients had received TIVA with propofol as maintenance of general anesthesia during the specified period, and 596 patients received sevoflurane for maintenance anesthesia. Records from 5 patients were missing. After matching patients in SEVO group with patients in the TIVA group, there were 95 patients in the SEVO group and 95 patients in the TIVA group. Patient demographics are summarized in Tables 1 and 2. Mean intraoperative remifentanil consumption was significantly higher in the TIVA group than in SEVO group (2369.36 vs 1735.94 μg, *P* < .001). In the SEVO group, there were no chronic pain patients and none of the patients were taking opioids preoperatively. There was 1 chronic pain patient who was on preoperative opioids in the TIVA group. There was no significant difference in the number of patients with chronic pain or those taking preoperative opioids between the 2 groups (both *P* = 1.000) (Table 1). There were no significant differences in other patient demographics. The mean intraoperative ketamine consumption was similar between the 2 groups [SEVO group 12.89 mg (SD 13.40) vs TIVA group 19.53 mg (SD 23.2), *P* = .114] (Table 2). The data were not normally distributed, and so Mann–Whitney test was used to look for differences between the 2 groups.

**Table 1:**
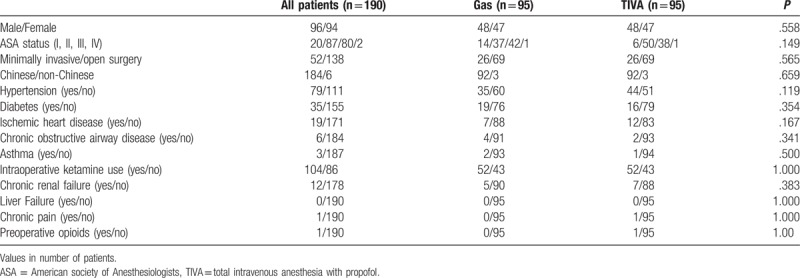
Patient demographics, duration of surgery, and total intraoperative opioid and ketamine consumption.

**Table 2:**
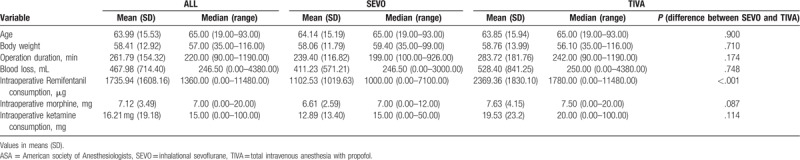
Patient demographics, duration of surgery, and total intraoperative opioid and ketamine consumption.

There were no significant differences in NRS pain score at rest or during coughing between the 2 groups (Table 3). In order to investigate whether there were any differences in pain scores when analyzing open surgery individually, a subgroup analysis was undertaken evaluating only patients undergoing open colorectal surgical procedures. There were also no significant differences in NRS pain scores (postoperative days 1–3) in this subgroup (data not shown).

**Table 3:**
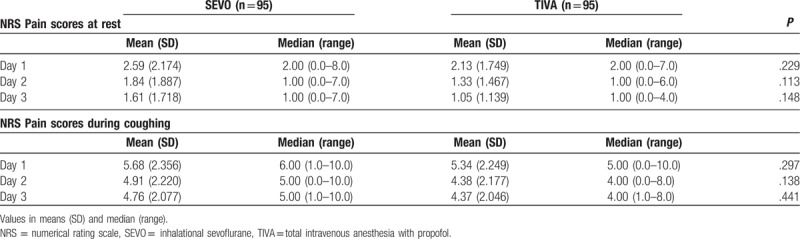
Postoperative pain scores.

Nonaccumulative daily PCA morphine consumption is summarized in Table 4. There was a significantly reduced daily mean morphine consumption in the TIVA group on postoperative days 1 to 3 compared with SEVO group (11.02 vs 14.12 mg, *P* = .032 on day 1, 8.26 vs 13.45 mg, *P* = .002 on day 2, and 4.22 vs 5.90 mg on day 3, *P* = .031). Total PCA morphine consumption over the 3 days was also significantly less in TIVA group compared with SEVO group (16.64 mg vs 24.07 mg, *P* < .001).

**Table 4:**
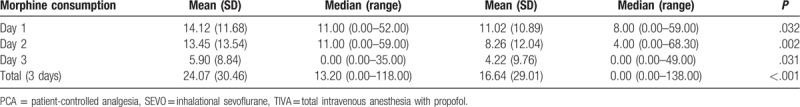
Nonaccumulative daily PCA morphine consumption (mg).

No differences were observed between the number of patients suffering from nausea, vomiting, dizziness, pruritus, and confusion between the 2 groups (Table 5). It should be noted that this study was not statistically powered to investigate the differences in adverse effects between groups; therefore, results shown here are exploratory only and the power to detect these side effects is low. There were no differences between groups regarding overall patient satisfaction with postoperative pain control (Table 6).

**Table 5:**
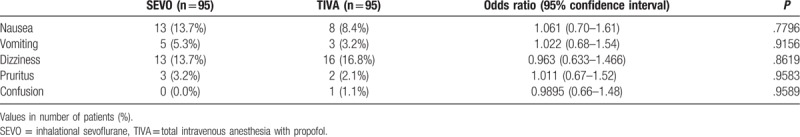
Postoperative side effects.

**Table 6:**
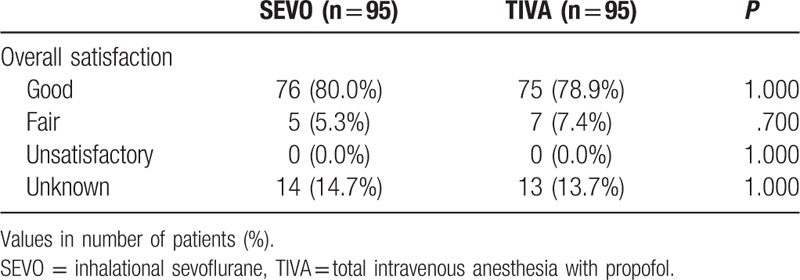
Overall satisfaction with postoperative pain control.

## 4 Discussion

In this study, we found no differences in postoperative pain scores, incidence of side effects, or patient satisfaction with pain control between patients anesthetized with TIVA propofol and those having inhalational sevoflurane. However, TIVA with propofol was associated with significantly reduced postoperative morphine consumption from postoperative days 1 to 3.

There were no significant differences in NRS pain scores both at rest and with coughing between TIVA group and SEVO group. Some other studies have also shown no difference in pain scores between inhalational anesthetics and TIVA with propofol.^[14,15]^ However, a meta-analysis of 14 randomized controlled trials showed that TIVA with propofol was associated with reduced postoperative NRS pain scores 24 hours after surgery compared with inhalational anesthesia, although difference was marginal.^[12]^ Another meta-analysis also showed reduced postoperative pain scores at rest 30 minutes, 1 hour, and 12 hours after surgery, although the difference was not significant when a conservative *P* value of less than .1 was used.^[30]^ Using a similar anesthetic/analgesic protocol to the one used in this current study, patients receiving TIVA with propofol had significantly lower NRS pain scores with coughing on postoperative days 1 to 2 after liver surgery.^[9]^ The difference observed between the 2 studies may be due to the nature of the surgical wounds. Liver surgery is associated with large upper abdominal wounds, which usually result in more postoperative pain than lower abdominal wounds, especially with coughing. Therefore, use of TIVA with propofol in that setting probably produced a relatively bigger difference in NRS pain scores. This suggests that the effectiveness of TIVA with propofol in reducing postoperative pain is procedure specific and dependent on the type of surgery being performed. Another reason why no differences in pain scores were found may be due to the effect of intraoperative remifentanil. Intraoperative remifentanil consumption was significantly higher in the TIVA group than in SEVO group in this current study (Tables 1 and 2). High-dose intraoperative remifentanil infusion induces acute opioid tolerance and opioid-induced hyperalgesia, and results in higher postoperative pain scores and opioid consumption.^[31–33]^ Acute tolerance and opioid-induced hyperalgesia may occur at infusion rates above 0.25 and 0.2 μg/kg/min, respectively.^[31]^ As this was a retrospective study, we were not able to control the amount of remifentanil used intraoperatively. Higher intraoperative remifentanil consumption may have resulted in higher NRS pain scores in TIVA group patients than would have otherwise occurred if they had received a similar amount of remifentanil to SEVO group patients.

TIVA with propofol was associated with significantly reduced total postoperative PCA morphine consumption, as well as daily PCA morphine consumption on postoperative days 1 to 3. Total PCA morphine consumption reduced by around 45% in the TIVA group, which is clinically significant. This is in agreement with findings from several clinical studies, which also found reduced postoperative opioid consumption in patients receiving propofol for maintenance anesthesia.^[8,9,34]^ A meta-analysis also showed that TIVA with propofol was associated with reduced morphine consumption at 24 hours, although this was not significant when using a conservative *P* value of .01.^[30]^ Meanwhile, other studies have not shown any significant difference in postoperative opioid consumption between TIVA with propofol and inhalational anesthesia.^[10,12,14,15]^ The reason for differences between studies may be due to differences in surgical procedures and also a difference in anesthetic/analgesic regimes. Use of intraoperative remifentanil may also influence whether TIVA with propofol is beneficial or not. Propofol reduces hyperalgesia caused by remifentanil infusion,^[35]^ and the analgesic effect of TIVA with propofol versus inhalational anesthesia has been shown to be better in patients receiving intraoperative remifentanil.^[30]^ This is thought to be due to propofol's inhibitory effect on NMDA receptors, which is a receptor that causes remifentanil-induced hyperalgesia.^[36]^ Patients in this retrospective study all received intraoperative remifentanil. Similarly, another retrospective study done by our group using a similar anesthetic and analgesic regime on patients undergoing liver surgery also showed a significantly lower opioid consumption in the TIVA group.^[9]^ Therefore, opioid-sparing effects of propofol may be more pronounced when intraoperative remifentanil is used.

Analgesic adjuvants that can reduce perioperative opioid consumption are important in colorectal surgery, especially in the context of ERAS programs. Reduced opioid consumption reduces bowel dysfunction and is associated with earlier recovery.^[18,19]^ In a study focusing on enhanced recovery pathways for laparoscopic colorectal surgery, low opioid intake (morphine equivalent of 30 mg or less) was one of the factors associated with early discharge within 48 hours.^[19]^ In this study, reduced postoperative morphine consumption was observed from postoperative days 1 to 3. A multimodal analgesic regime consisting of local wound infiltration and multiple oral analgesics was used in this study, which is in line with the concept of multimodal analgesia in ERAS. As TIVA with propofol was associated with reduced opioid consumption, it may potentially enhance recovery and reduce bowel dysfunction in patients undergoing colorectal surgery within a multimodal analgesic regime. However, information regarding recovery, hospital discharge, and return of bowel function were not available for analysis.

No differences between groups were noted for the incidence of side effects: nausea, vomiting, dizziness, confusion, and pruritus. In addition, there was no significant difference in patient satisfaction with pain control. TIVA with propofol has been shown to reduce nausea and vomiting compared with inhalational anesthesia.^[12,37]^ In our study, there was a trend toward reduced nausea in the TIVA group compared with SEVO group (8.4% vs 13.7%, *P* = .355), but this was not statistically significant. However, this study was not powered to detect significant differences in side effects, which have a low incidence.

A limitation of this study was that the data were retrospectively collected. Thus, not all variables could be controlled. Depth of anesthesia was not controlled nor monitored using depth of anesthesia monitoring. In addition, as mentioned earlier, intraoperative remifentanil consumption was significantly higher in the TIVA group, which could have affected the pain scores. Another limitation in this study was that some patients received open surgery, while others had minimally invasive surgery. These patients were matched equally between the TIVA and SEVO group. When analyzing the subgroup receiving open surgery, there was still no significant difference in pain scores, which was consistent with the results of the total combined data. In our analgesic regime, we employed a multimodal approach using a combination of local anesthetic wound infiltration, intravenous PCA morphine, and multimodal oral analgesics. However, regional techniques such as thoracic epidural analgesia were not performed, as thoracic epidural analgesia was not routinely used in our center for colorectal surgery. The opioid-sparing effect of TIVA with propofol demonstrated in this study may not be present when thoracic epidurals are used. Finally, we did not have information about the total dose of oral analgesics taken postoperatively. Although patients from both groups were prescribed regular paracetamol, celecoxib, and tramadol, we cannot be certain that all patients took the same amount of oral analgesic drugs. Therefore, we cannot rule out the possibility of significant differences in oral analgesic consumption between the 2 groups that may have affected the results of the study.

In conclusion, TIVA with propofol was not associated with improvements in postoperative pain scores, side effects, and patient satisfaction compared with inhalational sevoflurane in patients undergoing colorectal surgery. It was associated with significantly reduced postoperative PCA morphine consumption. Larger sample sized randomized controlled studies are needed to confirm the opioid-sparing effects of TIVA with propofol.

## Acknowledgment

The authors wish to thank Ms J. Man for input in statistical analysis, and The Department of Anaesthesiology, University of Hong Kong for supporting this project.

## Author contributions

**Conceptualization:** Stanley Sau Ching Wong.

**Data curation:** Siu Wai Choi, Yvonne Lee.

**Formal analysis:** Siu Wai Choi.

**Investigation:** Yvonne Lee.

**Methodology:** Stanley Sau Ching Wong, Siu Wai Choi.

**Supervision:** Stanley Sau Ching Wong, Chi Wai Cheung.

**Writing – original draft:** Stanley Sau Ching Wong, Siu Wai Choi.

**Writing – review & editing:** Stanley Sau Ching Wong, Michael Garnet Irwin, Chi Wai Cheung.
